# Quantitative estimation of pesticide-likeness for agrochemical discovery

**DOI:** 10.1186/s13321-014-0042-6

**Published:** 2014-09-12

**Authors:** Sorin Avram, Simona Funar-Timofei, Ana Borota, Sridhar Rao Chennamaneni, Anil Kumar Manchala, Sorel Muresan

**Affiliations:** 1 Department of Computational Chemistry, Institute of Chemistry of Romanian Academy Timisoara, 24 Mihai Viteazul Avenue, Timisoara, 300223 Romania; 2 GVK Biosciences Pvt. Ltd., S1, Phase-1, Technocrats Industrial Estate, Hyderabad, 500 037 Balanagar India; 3 Food Control Department, Banat's University of Agricultural Sciences and Veterinary Medicine, Calea Aradului 119, Timisoara, 300645 Romania

**Keywords:** Herbicide, Insecticide, Fungicide, Pesticide, Agrochemicals, SAR databases

## Abstract

**Background:**

The design of chemical libraries, an early step in agrochemical discovery programs, is frequently addressed by means of qualitative physicochemical and/or topological rule-based methods. The aim of this study is to develop quantitative estimates of herbicide- (QEH), insecticide- (QEI), fungicide- (QEF), and, finally, pesticide-likeness (QEP).

In the assessment of these definitions, we relied on the concept of desirability functions.

**Results:**

We found a simple function, shared by the three classes of pesticides, parameterized particularly, for six, easy to compute, independent and interpretable, molecular properties: molecular weight, logP, number of hydrogen bond acceptors, number of hydrogen bond donors, number of rotatable bounds and number of aromatic rings. Subsequently, we describe the scoring of each pesticide class by the corresponding quantitative estimate. In a comparative study, we assessed the performance of the scoring functions using extensive datasets of patented pesticides.

**Conclusions:**

The hereby-established quantitative assessment has the ability to rank compounds whether they fail well-established pesticide-likeness rules or not, and offer an efficient way to prioritize (class-specific) pesticides. These findings are valuable for the efficient estimation of pesticide-likeness of vast chemical libraries in the field of agrochemical discovery.

**Electronic supplementary material:**

The online version of this article (doi:10.1186/s13321-014-0042-6) contains supplementary material, which is available to authorized users.

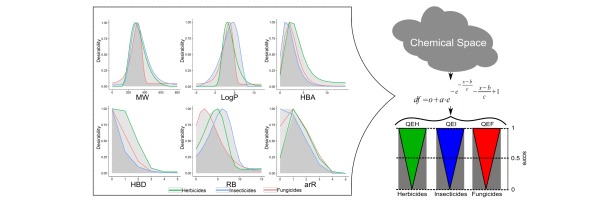

## Background

In the past years, the systematic identification of new lead compounds has gained increasing attention in both pharmaceutical and agrochemical industries. The progress of combinatorial chemistry (the parallel synthesis of large numbers of compounds) and high-throughput screening (the parallel testing for bioactivity of large numbers of compounds) facilitated the exploration of extensive chemical spaces for chemicals with desirable properties. In order to conduct effectively a drug/agrochemical discovery program, a screening library should contain compounds displaying reasonable properties to ease the passage to final products. Thus, in the early stages of such programs, *in silico* approaches are used to design chemical libraries [1],[2]. Oral bioavailability or membrane permeability have often been connected to simple molecular descriptors such as logP, molecular weight, or the counts of hydrogen bond acceptors and donors in a molecule [3]. Hence, over the years, simple rule-based models were derived based upon physicochemical and structural property of available datasets. These qualitative approaches (also referred to as filters) retain or reject molecules depending on a set of strict threshold values for key molecular descriptors (often combined with the presence or absence of undesirable chemical groups). This provides a rapid way to select molecules showing increased likelihood to exhibit the specific property for which the filter has been designed for [4]-[7].

In drug discovery, Lipinski's rule of five (Ro5) is considered to be the reference in defining physicochemical and structural properties profiles for optimal bioavailability of drug candidates [3]. Upper limits of five basic molecular descriptors were established based upon a set of known drugs, i.e., molecular weight ≤500, octanol/water partition coefficient (hydrophobicity) ≤5, number of hydrogen bond donors ≤5 and number of hydrogen bond acceptors ≤10. Molecules that would obey these rules should exert acceptable solubility and cell permeability properties and were defined as `drug-like' [3]. Although Ro5 is considered predictive for oral bioavailability, 16% of oral drugs violate at least one of the criteria and 6% fail two or more [8]. Other simplified rule-based definitions of drug-likeness were established by Veber [9] and Ghose [10].

In the field of agrochemical discovery, Lipinski's Ro5 approach was quickly adopted to profile agrochemicals, i.e., herbicides, insecticides and fungicides [11],[12]. In this sense, a referential paper was published by Tice [11], who defined, using Ro5 molecular descriptors, criteria to identify herbicides and insecticides, the two major classes of pesticides (see Table 1). Clarke & Delaney added further molecular properties known to influence absorption and distribution of agrochemicals, i.e., predicted solubility, melting point, ΔlogP, charge, acidity and basicity, percentage of aromatic atoms and non-carbon atoms [12]. In a more recent work Clarke [13] established upper limits of Abraham descriptors McGowan volume, hydrogen bond acidity and the hydrogen bond basicity. Investigating the constitutive properties of a representative library of marketed pesticides, from different periods of registration, Hao et al. [14] defined simple and easy to implement rules for pesticide-likeness, by including molecular weight (MW), hydrophobicity (LogP), number of H-bond acceptors (HBA) and donors (HBD), number of rotatable bonds (RB) and number of aromatic bounds.

**Table 1:** **Rule-based filters for drugs and pesticides**

Rule	Lipinski	Tice		Hao
Class	Drugs	Herbicides	Insecticides	Pesticides
MW	≤ 500	150 – 500	150 – 500	≤ 435
MLogP(*CLogP)	≤ 5	≤ 3.5	0 - 5	≤ 6*
HBD	≤ 5	≤ 3	≤ 2	≤ 2
HBA	≤ 10	2 - 12	1 – 8	≤ 6
RB	-	< 12	< 12	≤ 9
aromatic bonds	-	-	-	≤ 17

*MLogP [15] values were computed for Lipiniski's [3] and Tice's [11] rules and CLogP [16] values for Hao's [14] according to the original publications.

To overcome the hard boundaries established by traditional filters for drug-likeness, Bickerton et al. [8] developed the so-called quantitative estimate of drug-likeness (QED) which combines the simplicity of rules-based methods and the ranking advantages of continuous models. The approach relies on a small number of relevant, accessible and quick to compute, molecular descriptors describing the distribution of a set of molecules. So-called desirability functions [17], i.e., functions that describe the distribution of the data, have been fitted for each descriptor. Hence, QED defines drug-like molecules on a continuous scale, ranging from zero (the least drug-like) to one (the most drug-like) [8].

We consider that the field of agrochemical discovery would benefit from a similar treatment of pesticide-likeness. Thus, in this study, we aim to establish quantitative estimates of pesticide-likeness. Three main classes of pesticides are considered herein, i.e., herbicides, insecticides and fungicides, and, accordingly, we describe the quantitative estimate of herbicide-likeness (QEH), of insecticide-likeness (QEI) and of fungicide-likeness (QEF). We found a simple type of function that accurately describes six physicochemical properties over the three pesticide classes. Furthermore, we compare the performance of this quantitative approach to well known rule-based methods defining pesticide-likeness using a large library of patented compounds for agrochemical applications and discuss the results. For practical reasons and for the purpose of this paper, we will denominate the ensemble of scoring functions dedicated to pesticide-likeness as QEPest-SFs.

## Results and discussion

### The assessment of a common desirability function for pesticides

We applied the concept of desirability [17] to provide a quantitative metric for assessing pesticide-classes-likeness and subsequently pesticide-likeness. The desirability function approach was originally proposed by Harrington [17] and later refined by Derringer and Suich [18]. The approach consists of employing one/several functions to characterize the properties of several dependent variables, normalize (scale between zero and one) and combine the resulted terms using the geometric mean. Since we deal with molecular data sets, we followed the procedure of Bickerton's et al. [8] which derived series of desirability functions, each for a different molecular descriptor.

Here, we sought to find a type of function (as simple as possible) that would accurately fit distributions resulted from molecular properties describing herbicides, insecticides and fungicides. Firstly, we computed a number of 15 molecular descriptors (see Additional file 1: Table S1) for the 1685 marketed pesticides (see *Marketed pesticide set* section in Methods). The resulted distributions of the three pesticide-classes were fitted as described in *Curve fitting* section in Methods. We found six independent (see Additional file 1: Figure S1) molecular descriptors, closest to those enumerated in Table 1 showing adequate distribution of data and accurate fitting curves (for the three pesticide classes), i.e., MW, LogP, HBA, HBD, RB and arR (number of aromatic rings). We examined the first fifty equations ranked, increasingly, according to the lowest sum of squared absolute error, as computed by the fitting algorithm. Accordingly, we selected the function showing the smallest sum of ranks among the three classes of pesticides. Thus, a simple function *f* (eq. 1) was selected, parameterized by *o*, *a*, *b*, *c*, coefficients computed for each distribution of pesticide-class and molecular descriptor (see Additional file 1: Table S2).

1 $$f=o+a\cdot e^{-e^{\frac{-x-b}{c}}\;-\;\frac{x-b}{c}\;+\;1}$$

**Figure 1:**
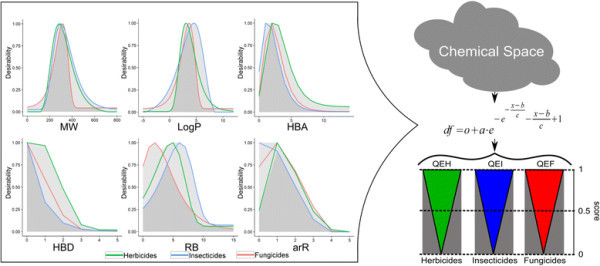
**Frequency counts and desirability function plots of herbicides.** Histograms and desirability functions (red curve, see right scale) of six molecular descriptors, i.e., MW (molecular weight), LogP (log of the octanol–water partition coefficient), HBA (number hydrogen bond acceptors), HBD (number hydrogen bond donors), RB (number of rotatable bonds), arR (number of aromatic rings) computed for the herbicides subset.

**Figure 2:**
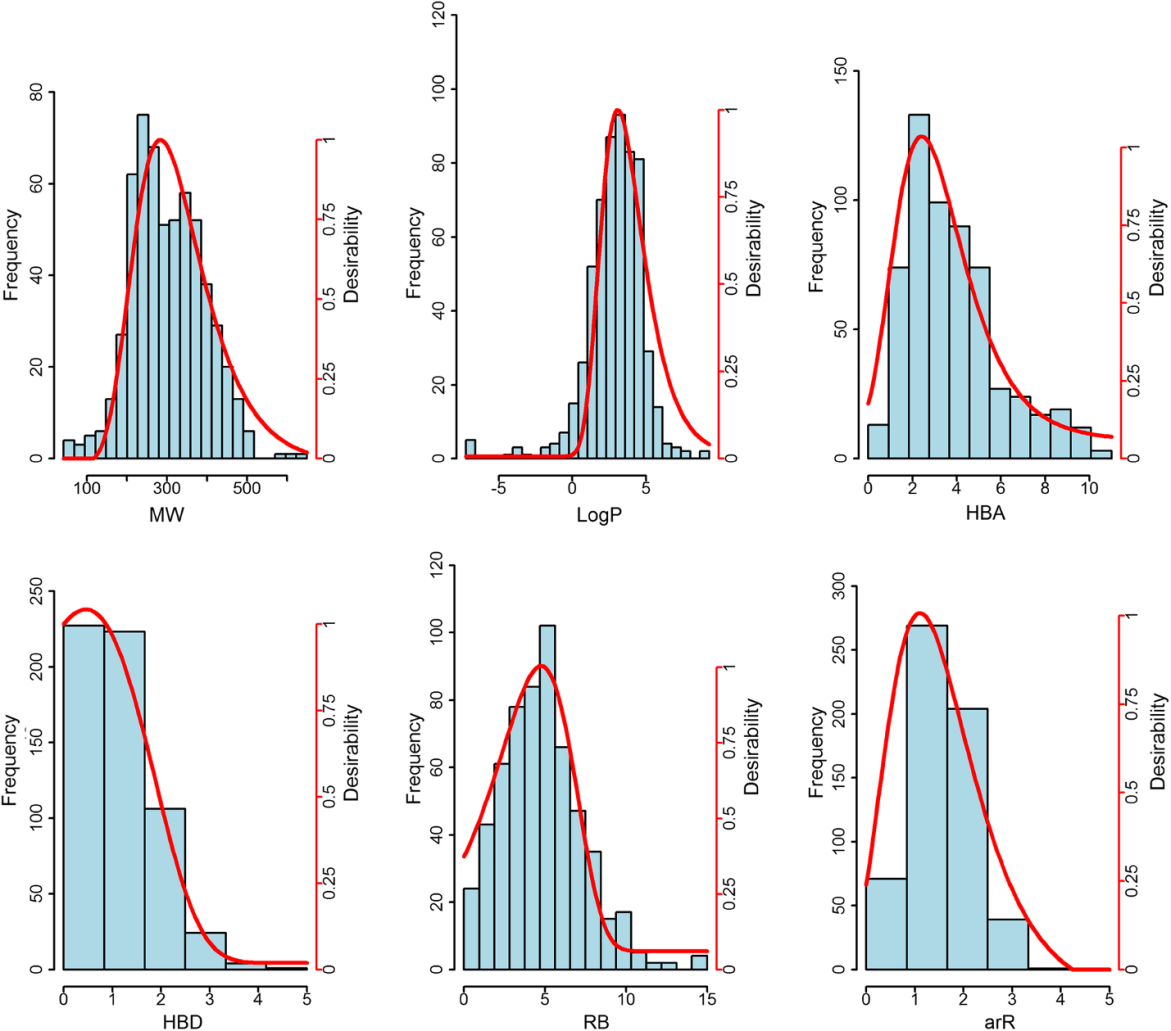
**Frequency counts and desirability function plots of insecticides.** Histograms and desirability functions (red curve, see right scale) of six molecular descriptors, i.e., MW (molecular weight), LogP (log of the octanol–water partition coefficient), HBA (number hydrogen bond acceptors), HBD (number hydrogen bond donors), RB (number of rotatable bonds), arR (number of aromatic rings), computed for the insecticides subset.

**Figure 3:**
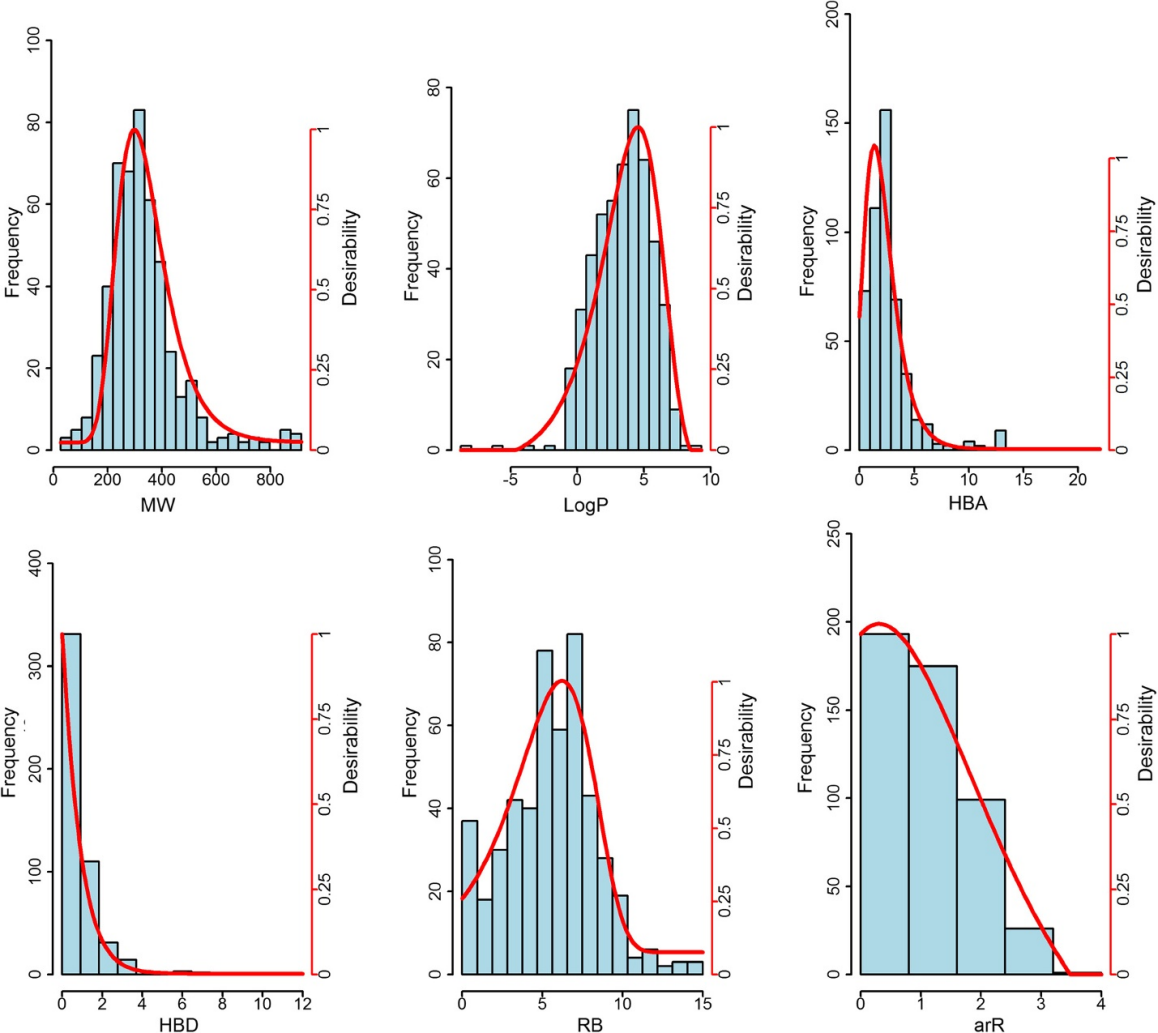
**Frequency counts and desirability function plots of fungicides.** Histograms and desirability functions (red curve, see right scale) of six molecular descriptors, i.e., MW (molecular weight), LogP (log of the octanol–water partition coefficient), HBA (number hydrogen bond acceptors), HBD (number hydrogen bond donors), RB (number of rotatable bonds), arR (number of aromatic rings), computed for the fungicides subset.

In order to assure reasonable desirability scores, function *f* was scaled between zero and one by division with maximum values (see Additional file 1: Table S2). Thus, the value of the resulted desirability function *df*, increases as the 'desirability' of the corresponding response increases (see Figures 1, 2 and 3). The accuracy of the fittings is reported in Additional file 1: Table S3.

The individual *df*_*i*_ (*i* molecular descriptor) were joined accordingly for each pesticide-class by computing geometric means. This can be expressed by logarithmic identities, as the exponent of the arithmetic mean of the logarithm transformed *df*s (see eq. 2). As argued by Derringer and Suich [18] the geometric mean exhibits several advantages in this case: (*i*) zero to one range, (*ii*) output values will increase as the balance of the properties becomes more favorable, (*iii*) if any *df*_i_ = 0 (is unacceptable) the geometric mean is null, i.e., if a property is unacceptable the compounds becomes unacceptable.

2 $$\mathrm{QEX}=e^{\;\frac{1}{n}\;\overset{n}{\underset{i=1}{\Sigma }}\ln df_{i}},\mathrm{for}\;df_{i}>0;\mathrm{if}\;df_{i}\leq 0,\mathrm{QEX}=0,\mathrm{where}\;X=\left\{"H","I","F"\right\}$$

We denominate the resulted scoring functions as quantitative estimates of herbicide-likeness (QEH), insecticide-likeness (QEI) and fungicide-likeness (QEF), according to the pesticide class. These functions reflect the probability of a molecule to exhibit desirable characteristics as a pesticide. Thereby, we obtained an intuitive quantitative indicator of the likeness of a molecule to match the physicochemical profile of pesticides.

In order to model specific properties of large data sets, predictive models often use many descriptors limiting the applicability domains of the model. The more descriptors are used, the greater is the likelihood that a candidate molecule will fall outside the limits of one or more of these descriptors [19]. In our approach, we limit the number of descriptors to six basic physicochemical, independent, properties, correlated with pesticide bioavailability, solubility and stability [3],[9],[20],[21]. These descriptors are included also in the formulation of QED [8] to define drug-likeness, and moreover, with a slight variation, i.e., count of aromatic rings – arR – replaced by count of aromatic bonds, the same properties were are encountered in Hao's [14] approach to identity pesticides (see Table 1).

### Pesticide class scorings

The three main classes of pesticides are: herbicides (against weeds), insecticides (against harmful insect pests), and fungicides (against harmful diseases) [12],[14],[22]. In this section, we will describe the way the above established pesticide class-specific desirability functions relate to each other.

**Figure 4:**
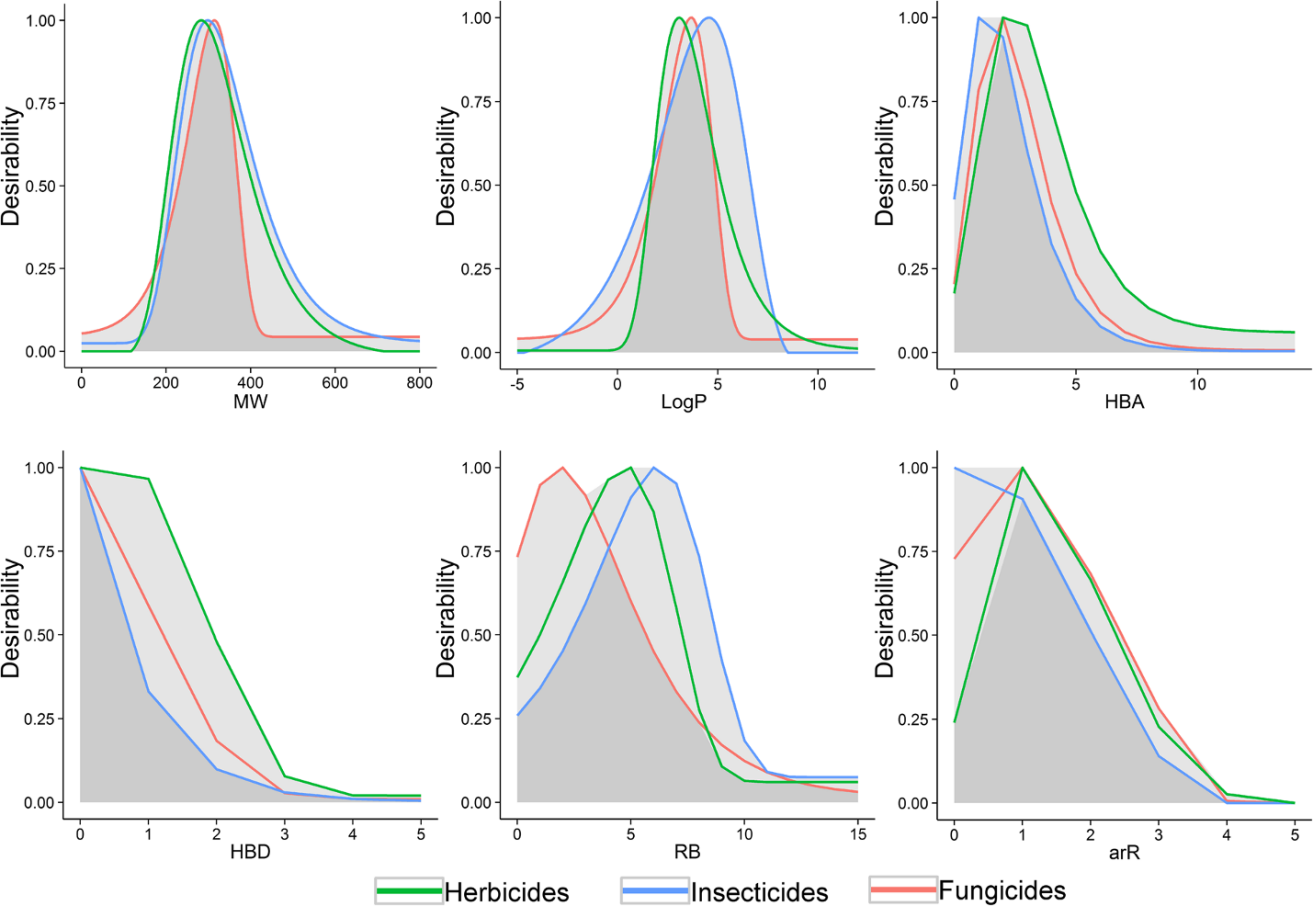
**Comparative representation of desirability functions.** Desirability function curves describing the three classes of pesticides: herbicides, insecticides and fungicides, in terms of MW (molecular weight), LogP (log of the octanol–water partition coefficient), HBA (number hydrogen bond acceptors), HBD (number hydrogen bond donors), RB (rotatable bonds), and arR (number of aromatic rings); dark grey –overlapping area described by the three curves; light grey – maximum area described by the three curves.

In Figure 4 we plotted herbicide, insecticide and fungicide desirability functions against each variable separately. Differences between the three classes can be observed for all descriptors. In the case of MW ranging between 400 and 500, herbicides and insecticides can receive considerable higher scores compared to fungicides. One can observe that insecticides span over a broader range of LogP values. A considerable drop in scoring herbicides and fungicides can be noted at LogP > 5.5, whilst insecticides reach maximal desirability around this LogP value. The more hydrophilic nature of herbicides (and fungicide), in comparison to insecticides, is further consistently underlined in the HBA and HBD plots. More noticeable differences are present in the number of rotatable bounds plot: the peaks of the functions are reached at 2 RB for fungicides, 5 RB for herbicides and 6 RB for insecticides, but considerable area overlap can be observed. Finally, non-aromatic molecules provide major scoring variations between pesticide-classes: herbicides are poorly scored and, in contrast, insecticides gain maximum desirability scores.

The recent analysis, conducted by Hao et al. [14], concerning the distributions of herbicides, insecticides and fungicides as described by six molecular descriptors, i.e., MW, ClogP, HBA, HBD, RB, number of aromatic bonds, indicated CLogP, HBD, and the number of aromatic bonds to be important constitutive properties to distinguish between the three classes of pesticides. Furthermore, the same study, describes RB distributions of herbicides and fungicides to be similar, with lower values compared to insecticides [14]. We note that, for the most part, our *df*s agree with previous findings, and slight variations in the distributions might be reasoned by the various datasets employed.

## Experimental

### AgroSAR patent database

GVKBio agrochemical patents collection (AgroSAR) comprises ~ 59 k (58915) unique structures and ~ 413 k (413103) SAR end-points measured in ~110 k (109733) assays. A percentage of 38.7% of the data has been published in the seventies, 29.6% in the eighties and 28.67% in the nineties up to 2005. AgroSAR gathers herbicides, insecticides, fungicides, acaricides, nematocides, bactericides, algaecide, plant growth, biocides, microbiocides and rodenticides in a relational database, manually curated and annotated, easy to query and subset. This database comprises large amounts of unexplored patent data, which can help to improve the discovery of agrochemicals. To our knowledge, this is the only SAR patent database built specifically from patent specifications filed in the agro sector.

We selected a subset of potent herbicides, insecticides and fungicides available in AgroSAR, as defined by more than 50% activity obtained at concentrations of 4.5 lb/acre (0.826 kg/ha) for herbicides, 125 ppm for insecticides and 100 mg/L for fungicides (cutoffs established by the medians of the activity data available per class). Hence, after removing marketed pesticides, we retrieved 1105 herbicides, 8983 insecticides and 9371 fungicides (Table 2). In this study, we will employ only these sets to assess the pesticide-likeness by various methods.

**Table 2:** **Pesticide sets extracted from AgroSAR**

Class	Herbicides	Insecticides	Fungicides	Pesticides
Num. of compounds	1105	8983	9371	19459
Ro5 (%)	97.29%	73.56%	91.55%	83.65%

The class of Pesticides comprises compounds merged from the Herbicide, Insecticides and Fungicides sets; Ro5 (%) - percentages of compounds passing Lipinski's Ro5 with no violation.

**Figure 5:**
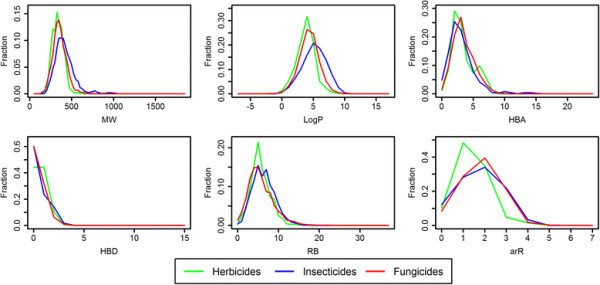
**Basic molecular properties of herbicides, insecticides and fungicides selected from AgroSAR.** Comparative distribution plots of AgroSAR selected herbicides (green), insecticides (blue) and fungicides (red), in terms of MW (molecular weight), LogP (log of the octanol–water partition coefficient), HBA (number hydrogen bond acceptors), HBD (number hydrogen bond donors), RB (rotatable bonds), and arR (number of aromatic rings).

Basic statistics to describe the AgroSAR database are reported in Table 3 (and individual statistics of pesticide-class sets are reported in Additional file 1: Table S4). Additionally, a graphical description of the pesticide class-distributions in AgroSAR is shown in Figure 5. One can observe a slight shift towards higher molecular weight and LogP values in the case of insecticides compared to fungicides and herbicides. The latter two seem to exhibit more similarities, however, in term of arR, most herbicides display a smaller number of aromatic rings compared to insecticides and fungicides.

**Table 3:** **Statistics of the pesticides extracted from AgroSAR**

Properties	5% quantile	95% quantile	Median	Mean	SD
MW	228.3	553.3	354.8	370.1	108.2
LogP	1.2	7.2	4.1	4.2	1.8
HBA	1	7	3	3.3	2
HBD	0	2	0	0.5	0.8
RB	2	11	6	6.1	3.1
arR	0	3	2	1.8	1

SD - standard deviation; MW - molecular weight; LogP - hydrophobicity; HBA - number of hydrogen bond acceptors; HBD - number of hydrogen bond donors; RB - number of rotatable bonds; arR - number of aromatic rings.

**Figure 6:**
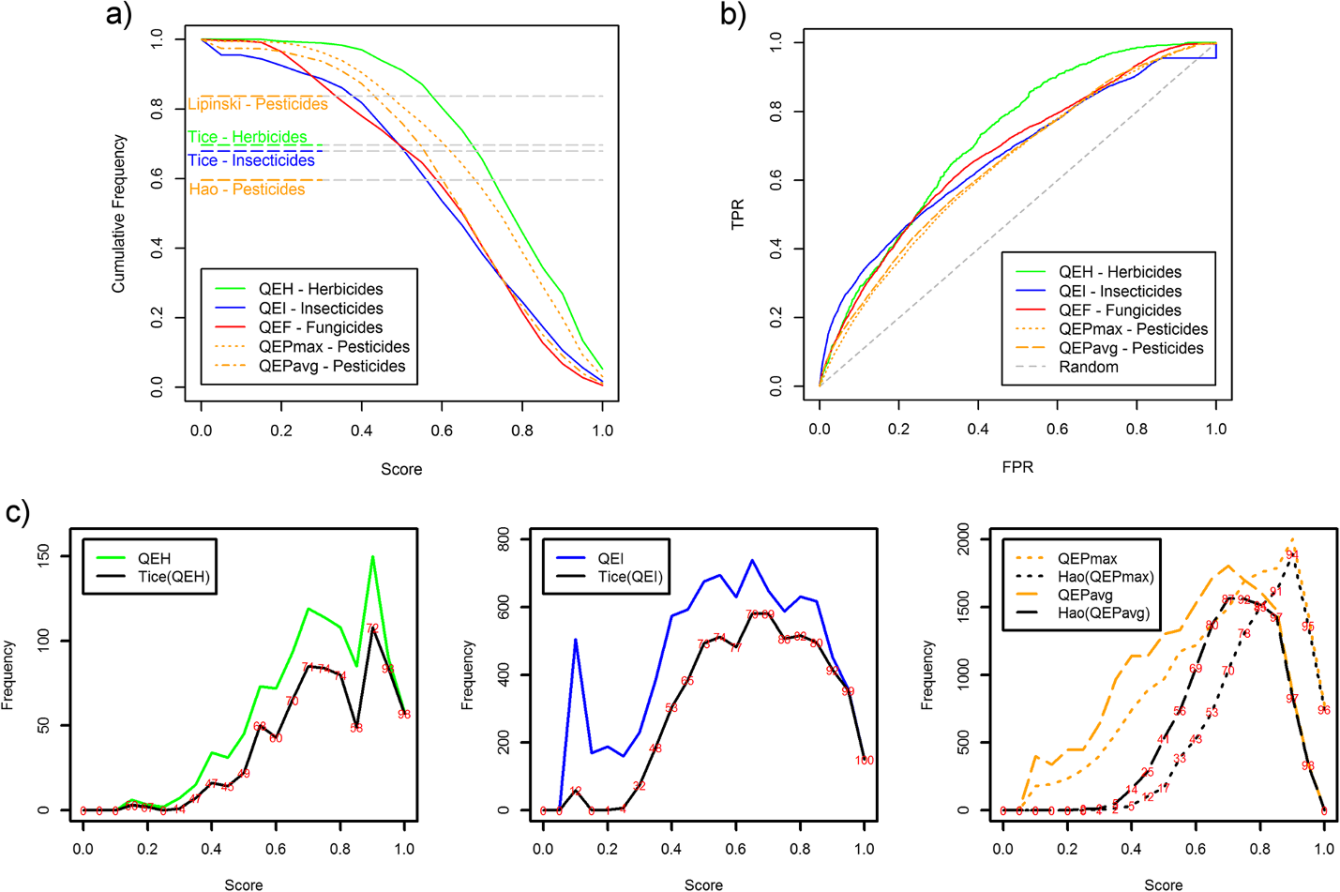
**Evaluation of AgroSAR pesticides. (a)** Cumulative frequencies of AgroSAR pesticide sets (herbicides – green, insecticides – blue, fungicides – red, pesticides – orange) plotted against quantitative estimates scores and performance of Tice's, Hao's and Lipinski's rule-based approaches as describes in Table 1 (rule-type performances are represented independent from the x-axis score values) **(b)**; ROC curves showing the discriminative power of the scoring functions **(c)**; frequency distributions of herbicides (left), insecticides (middle) and pesticides (right) in terms of quantitative estimates scores and frequencies corresponding to compounds passing rule-based models (in red percentages of compounds passing rule-based filters *per* cutoff). In the panels: QEH, Quantitative estimate of herbicide-likeness; QEI, Quantitative estimate of insecticide-likeness; QEF, Quantitative estimate of fungicide-likeness; QEP, Quantitative estimate of pesticide-likeness; QEPmax and QEPavg, - the maximum and the average of QEH, QEI and QEF values, respectively.

Rule-based methods are widely used in the field of agrochemicals to identify chemicals with desirable properties. Based on a minimum set of easy-to-compute and interpretable molecular descriptors, we recall the efforts of Tice [11] and, more recently, Hao [14] to define herbicide- and insecticide-likeness and pesticide-likeness, respectively, as shown in Table 1. We evaluated the AgroSAR database, correspondingly, by means of these rules. We found that a percentage of 69.68% of the AgroSAR herbicides pass Tice's filter for herbicides (with zero violations) and 67.96% of AgroSAR insecticides pass Tice's filter for insecticides (with zero violations). We merged the AgroSAR pesticide-classes and applied Hao's rules for pesticide-likeness. The results indicate that 59.61% of the molecules are recognized (passed with no violation) as pesticides (Figure 6a).

The field of drug discovery is closely related to that of agrochemical-discovery. The development of new medicine offered by agrochemicals and *vice-versa* may benefit upon the similarities between agrochemical and pharmaceutical research [22]. Similar to drugs, modern-day pesticides are optimized for low mammalian toxicity and act *via* a single target at *nano*-molar concentrations. Herbicides and fungicides were reported to generally meet the Lipinski's Ro5 criteria for drug-like compounds [12]. This observation is strongly confirmed also by AgroSAR pesticide database: 97.29% of the herbicides and 91.55% of the fungicides pass Ro5 (with zero violation). In the case of insecticides, 73.56% of the molecules were recognized as drug-like (Table 2). We encountered similar results also for the marketed pesticide set (see Additional file 1: Table S5). As described above, insecticides exhibit a slightly different profile, compared to herbicides and fungicides, mainly consistent with increased hydrophobicity. Future explorations of these datasets can significantly contribute to improve the pesticide discovery and development programs.

### Scoring AgroSAR pesticide database

In this section, we will report and discuss the capabilities of the hereby-proposed scoring functions to quantitatively define pesticide-likeness. In addition to the quantitative estimates of class-specific pesticide-likeness, we explored two data fusion rules to provide quantitative estimates of pesticide-likeness. Hence, we define QEP_max_ and QEP_avg_, as the maximum and the average, respectively, of QEH, QEI and QEF values. The two fusion rules use QEH, QEI and QEF outcomes in different manners, i.e., the `max-value'- rule reflects only the highest pesticide-class score whilst the `average-value'-rule takes into account the contribution of all pesticide classes averaging the scores. Thus, in this section we will evaluate AgroSAR pesticides by means of QEH, QEI, QEF, QEP_max_ and QEP_avg_.

In Figure 6a, we show the cumulative frequency counts of herbicides, insecticide, fungicides and pesticides plotted against the scores assigned by the corresponding quantitative estimate function, i.e., QEH - herbicides, QEI - insecticides, QEF - fungicides, QEP_max_ - and QEP_avg_ - pesticides. The highest scores can be observed in the case of QEH scoring herbicides. According to the pesticide-class, half of the molecules received QEH scores ≥0.72 (herbicides), QEI scores ≥0.57 (insecticides), QEF score ≥0.6 (fungicides), QEP_max_ ≥0.7 and QEP_avg_ ≥0.6 (pesticides). These results, further supported by the cutoff values corresponding to 25% and 75% of the datasets (see Additional file 1: Table S6), confirm the ability of the scoring functions to assign high scores to the equivalent pesticide-class.

In Figure 6c, we show the distribution of herbicides, insecticides and pesticides against the corresponding scoring functions values, i.e., QEH, QEI, QEP_max_ and QEP_avg_. In order to see how these scores relate to well known rule-based models we plotted, correspondingly, the frequency counts of molecules passing Tice's filters for herbicides and insecticides, and Hao's filter for pesticides. One can observe a consistent trend between higher scores and increased percentages of compounds passing rule-based filters (Figure 6c).

**Figure 7:**
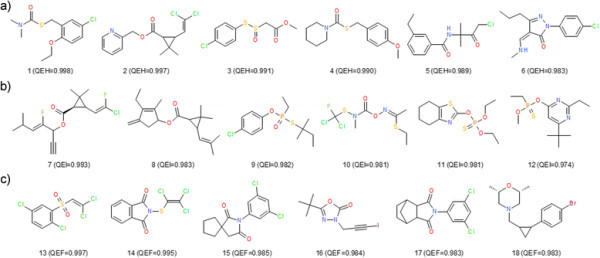
**Examples of highly scored AgroSAR pesticides.** Chemical representation of AgroSAR herbicides **(a)**, insecticides **(b)** and fungicides **(c)** and quantitative estimation scores in parenthesis.

To be marketed as pesticides, candidates need to meet a series of criteria, which cannot be fully addressed by the six molecular descriptors employed in QEPest-SFs. A number of 406 insecticides, 31 fungicides and 37 pesticides received null scores by the corresponding QEPest-SFs. On the other side, Figure 7, shows the chemical representation of the six best scored herbicides, insecticides and fungicides in AgroSAR database. One can observe the more hydrophobic insecticides and also the abundance of halogens (more noticeable for the exemplified fungicides) underlines the observation of Jeschke P [23] according to which modern agrochemicals tend to be more halogenated. The equivalently poorest scored molecules (ignoring zero scored representatives) fall clearly outside the acceptable limits of most scoring functions (see Additional file 1: Figure S2) and were scored consequently.

Simple rule-based methods that define pesticide-likeness are applied in the early stages of pesticide-discovery programs. Due to their simplicity, these methods serve to trim large chemical libraries to smaller sets, which are supplied to more computational-expensive approaches. In this sense, a challenging exercise for QEPest-SFs would be to recognize pesticides from a larger set of decoys. In consequence, ten times larger sets of randomly chosen representatives from PubChem Compounds (http://pubchem.ncbi.nlm.nih.gov/; 46.75 million molecules downloaded on December 10, 2013) were assembled for each pesticide class. Using the same six molecular properties, we computed QEH, QEI, QEF, QEP_max_ and QEP_avg_ also for the decoys sets (the decoys assembled for the pesticide-classes were merged for the evaluation of QEP_max_ and QEP_avg_).

In Figure 6b, we show the ROC (receiver operating curve [24] – see *Performance measure* section in Methods) plots describing the capacity of QEH, QEI, QEF, QEP_max_ and QEP_avg_ to recognize the corresponding pesticide sets. A barely increased early enrichment can be seen in the case of QEI retrieving insecticides and, in contrast, QEH retrieved more lately herbicides. The discriminative performance was numerically assessed by AUC (area under the ROC [25] – see *Performance measure* section in Methods) values as reported in Additional file 1: Table S7. With the exception of QEH (AUC > 0.7), we encountered relative poor separation capabilities. However, these functions are not meant to be as accurate as virtual screening tools but rather estimative indicators of compounds showing desirable pesticide-like physicochemical properties. Moreover, the decoys employed here were not experimentally demonstrated to not qualify as pesticides. Thus, these results must be seen in the light of the purpose and utility of the scoring functions as described above.

QEPest-SFs have the ability to rank compounds whether they fail pesticide-likeness rules or not. In consequence, different cutoffs for the scoring functions provide various levels of sensitivity and specificity. One might be tempted to find optimal cutoffs values for these scoring functions. The results of such an approach are reported in Additional file 1: Table S8 and Figure S3. However, as underlined by Bikerton et al. [8] in the case of QED, the usage of any threshold is discouraged as this results in qualitative outcomes, similar to rule-based approaches. A practical application of the hereby-proposed scoring functions would be to rank compounds by their scores and select the number of top ranking compounds required.

## Conclusions

In this study, we have demonstrated that QEPest-SFs are able to rank compounds according to their herbicide-, insecticides-, fungicide- or pesticide-likeness. These scoring functions are based upon six simple molecular descriptors and a single type of function, parameterized accordingly to provide desirability scores. These quantitative assessments provide increased flexibility compared to traditional rule-based methods. For example, large chemical libraries can be reduced to desirable sizes, profiling pesticide-like molecules at various levels. In the usual pipeline of a drug and agrochemical discovery programs the resulted sets are supplied to more accurate virtual screening methods to increase cost-effectiveness in further experimental steps. For this purpose, we provide a simple Java-based program ("QEPest.jar") to compute QEH, QEI and QEF (see Additional file 2).

## Methods

### Marketed pesticide set

A set of 1685 pesticides (585 herbicides, 495 insecticides and 278 fungicides) was assembled from The Pesticide Manual [26] and Compendium of Pesticide Common Names [27]. For standardization (structure canonicalization and transformation – see Additional file 1: Table S9) the molecules were supplied to ChemAxon's Standardizer module (JChem 6.0.0, 2013, ChemAxon, http://www.chemaxon.com). The marketed pesticide set was used to derive quantitative estimate scoring functions for herbicide-likeness (QEH), insecticide-likeness (QEI), fungicide-likeness (QEF) and overall pesticide-likeness (QEP).

### Molecular descriptors

Molecular descriptors were computed with ChemAxon's structure database management software Instant JChem (JChem 6.0.0, 2013, ChemAxon, http://www.chemaxon.com). Six descriptors, i.e., molecular weight (MW), molecular hydrophobicity (log of the octanol–water partition coefficient; LogP), number of hydrogen bond acceptors (HBA), number of hydrogen bond donors (HBD), rotatable bonds (RB), aromatic rings (arR) were used to derive desirability functions for QEPest-SFs. Other hydrophobicity estimation metrics such as MLogP [15] and ClogP [16] were computed with Dragon (for Windows, Software for Molecular Descriptor Calculations, version 5.5, 2007 Talete srl, http://www.talete.mi.it) and BioByte (ClogP for Windows, version 1.0.0, 1995, BioByte Corp., http://www.biobyte.com/), respectively, and were used accordingly, as required by rule-based methods (Table 1).

### Distribution of data

For the assessment of the desirability functions we computed the frequency counts for each class of pesticides, according to the descriptor type-values, i.e., for continuous values (MW and LogP) the optimum bin size was computed with *Web Application for Bin-width Optimization* - Ver. 2.0 (http://176.32.89.45/~hideaki/res/histogram.html, accessed on Sep 21 2013) [28], and for discreet values (HBA, HBD, RB, arR) we used a bin-size of one (R 2.14.2) [29].

### Curve fitting

The frequency counts and bins computed for each molecular descriptor served as input for curve fitting processed by means of ZunZun.com *Online Curve Fitting and Surface Fitting Web Site* (http://zunzun.com/, accessed on Aug 6, 2013). Depending on the data to be modeled, up to 573 non-linearly, and 23 linearly equations, were fitted.

### Performance measure

The discriminative power of QEPest-SFs was assessed graphically and numerically by means of receiver operating curve (ROC) [24] and the area under the ROC (AUC) [25]. The ROC plot describes the true positive rate (TPR = sensitivity) *versus* the false positive rate (FPR = 1- specificity) according to the ranked list. AUC values indicate the ability of a scoring method (or prediction models, in general) to discriminate between two classes of elements, e.g., actives and inactives, and is defined by the area under the ROC. Values range from 0 to 1 (perfect separation), 0.5 suggesting a random spread of the representatives of the two classes.

## Authors' contributions

SM initiated and supervised the project. SA carried out the calculations, implemented and tested the scoring functions, developed the Java-based program and prepared the manuscript. SFT and AB contributed to data preparation for model development and validation and drafted the manuscript. SRC and AKM provided the AgroSAR patent database and corresponding annotations. All authors read and approved the final manuscript.

## Additional files

## Electronic supplementary material

Additional file 1: **Supporting Tables and Figures.** This pdf file contains nine tables **(** **Table S1's9** **)** and three figures **(** **Figure S1-S3** **)** offering supporting data as referenced throughout the paper. (PDF 396 KB)

Additional file 2: QEPest Java program. In the archive QEPest.zip we provide a simple Java-based program ("QEPest.jar") to compute QEH, QEI and QEF, based on pre-generated descriptors, accompanied by a input example ("data.txt"), an output file ("data.txt.out") and a "readme.txt" file for instructions. (ZIP 7 KB)

Below are the links to the authors’ original submitted files for images.

Authors’ original file for figure 1

Authors’ original file for figure 2

Authors’ original file for figure 3

Authors’ original file for figure 4

Authors’ original file for figure 5

Authors’ original file for figure 6

Authors’ original file for figure 7

Authors’ original file for figure 8

## Abbreviations

QEH: Quantitative estimate of herbicide-likeness
QEI: Quantitative estimate of insecticide-likeness
QEF: Quantitative estimate of fungicide-likeness
QEP: Quantitative estimate of pesticide-likeness
