# Supplementary material for: Quantitative estimation of pesticide-likeness for agrochemical discovery
Source: J Cheminform. 2014 Sep 12;6:42. doi: 10.1186/s13321-014-0042-6 (PMC4173135; doi:10.1186/s13321-014-0042-6)
Supplement: Supplementary file 1 — Additional file 1: Supporting Tables and Figures. This pdf file contains nine tables ( Table S1's9 ) and three figures ( Figure S1-S3 ) offering supporting data as referenced throughout the paper. (PDF 396 KB) [file 13321_2014_42_MOESM1_ESM.pdf]

Accessible quantitative estimation of pesticide-likeness for agrochemical discovery

Sorin Avram[1], Simona Funar-Timofei[1], Ana Borota [1], Sridhar Rao Chennamaneni[2], Anil Kumar Manchala[2], Sorel Muresan[3,§]

[1]Department of Computational Chemistry, Institute of Chemistry of Romanian Academy Timisoara, 24 Mihai Viteazul Avenue, 300223 Timisoara, Romania

[2]GVK Biosciences Pvt. Ltd., S1, Phase-1, Technocrats Industrial Estate, Balanagar, Hyderabad, 500 037, India

[3]Food Control Department, Banat's University of Agricultural Sciences and Veterinary Medicine, Calea Aradului 119, 300645 Timisoara, Romania

Contents

TABLE S1 MOLECULAR DESCRIPTORS (GENERATED WITH INSTANT JCHEM) EVALUATED FOR DESIRABILITY FUNCTIONS ASSESSMENT 2

TABLE S2 COEFFICIENTS AND MAXIMUM VALUES USED TO COMPUTE PROPERTY-SPECIFIC DESIRABILITY FUNCTIONS (*DF*S) FOR HERBICIDES, INSECTICIDES AND FUNGICIDES 3

TABLE S3 R^2 VALUES OF THE FITTED DESIRABILITY FUNCTIONS FOR THE THREE CLASSES OF PESTICIDES 4

TABLE S4 STATISTICS OF PESTICIDES EXTRACTED FROM AGROSAR SETS 4

TABLE S5 PERCENTAGES OF MARKETED PESTICIDES USED TO DEVELOP QEPEST-SFS PASSING RULE-BASED FILTERS 5

TABLE S6 QEPEST SCORES ASSIGN TO AGROSAR SETS AT CUMULATIVE FREQUENCY (CF) VALUES OF 0.25, 0.5, 0.75 5

TABLE S7 AREA UNDER THE RECEIVER OPERATING CURVE (AUC)[3] AND STANDARD DEVIATION (SD) VALUES ACHIEVED BY THE QEPEST SCORING FUNCTIONS 5

TABLE S8 OPTIMUM CUTOFFS FOR QEPEST-SFS AS SUGGESTED BY MATTHEWS CORRELATION COEFFICIENTS AND SENSITIVITY-SPECIFICITY *VERSUS* CUTOFF (SE-SP CUTOFF) PLOTS 6

TABLE S9 STANDARDIZATION OF PESTICIDES BEFORE MOLECULAR PROPERTIES GENERATION; 6

FIGURE S1 KENDALL *TAU* [4] VALUES DESCRIBING THE PAIR WISE CORRELATION BETWEEN MW, LOGP, HBA, HBD, RB AND ARR, IN THE MARKETED PESTICIDE SET (SEE PAPER) 7

FIGURE S2 THREE OF THE LOWEST SCORED HERBICIDES (A), INSECTICIDES (B) AND FUNGICIDES (C) FROM AGROSAR DATASET (IGNORING ZERO SCORED REPRESENTATIVES) 8

FIGURE S3 SENSITIVITY(SE)-SPECIFICITY(SP) AND MATTHEWS CORRELATION COEFFICIENT *VERSUS* SCORE CUTOFFS PLOTS OF AGROSAR (A) HERBICIDES, (B) INSECTICIDES, (C) FUNGICIDES, (D) PESTICIDES 9

§Corresponding author e-mail sorel.muresan@gmail.com

Table S1 Molecular descriptors (generated with Instant JChem) evaluated for desirability functions assessment

Number	Abbreviation	Molecular descriptor
1	AlphA	Aliphatic atom count
2	ArA	Aromatic atom count
3	ArB	Aromatic bond count
4	ArR	Aromatic ring count
5	chB	Side-chain bond count
6	HBA	Number of H-bond acceptors
7	HBD	Number of H-bond acceptors
8	LogP	Octanol/water particion coefficient
9	MW	Molecular mass
10	nC	Number of carbon atoms
11	nHl	Number of halogens
12	nHA	Number of heteroatoms
13	RB	Number of rotatable bonds
14	RC	Ring count
15	TPSA	Topological surface area

JChem 6.0.0, 2013, ChemAxon, http://www.chemaxon.com)

Table S2 Coefficients and maximum values used to compute property-specific desirability functions (*df*s) for herbicides, insecticides and fungicides

Pesticide class	Property	*a*	*b*	*c*	*o*	*Max*
Herbicides	MW	7.077E+001	2.830E+002	8.497E+001	-1.185E+000	69.5849922
	LogP	9.381E+001	3.077E+000	1.434E+000	6.164E-001	94.4228257
	HBA	1.176E+002	2.409E+000	1.567E+000	7.155E+000	120.4572352
	HBD	2.334E+002	4.535E-001	-1.480E+000	4.470E+000	228.1589796
	RB	8.470E+001	4.758E+000	-2.423E+000	5.437E+000	89.7012502
	arR	3.018E+002	1.101E+000	8.869E-001	-2.281E+001	276.9634213
Insecticides	MW	7.638E+001	2.983E+002	8.364E+001	1.912E+000	78.2919965
	LogP	7.427E+001	4.555E+000	-2.193E+000	-2.987E+000	71.2829691
	HBA	1.394E+002	1.363E+000	1.283E+000	5.341E-001	133.9224801
	HBD	6.706E+002	-1.163E+000	7.856E-001	7.951E-001	331.170104
	RB	6.549E+001	6.219E+000	-2.448E+000	5.318E+000	70.5540709
	arR	2.875E+002	3.050E-001	1.554E+000	-8.864E+001	193.0023343
Fungicides	MW	5.103E+001	3.142E+002	-5.631E+001	2.342E+000	53.3719946
	LogP	5.073E+001	3.674E+000	-1.238E+000	2.067E+000	52.773116
	HBA	7.379E+001	1.841E+000	1.326E+000	5.158E-001	73.7976536
	HBD	1.647E+002	-9.762E-001	-2.027E+000	1.384E+000	144.9887053
	RB	4.091E+001	1.822E+000	2.582E+000	6.235E-001	41.4385926
	arR	1.344E+002	8.383E-001	1.347E+000	-3.117E+001	102.3024319

Table S3 R^2 values of the fitted desirability functions for the three classes of pesticides

Class	MW	LogP	HBA	HBD	RB	arR
Herbicides	0.93	0.93	0.95	1.00	0.95	0.98
Insecticides	0.97	0.97	0.95	1.00	0.88	1.00
Fungicides	0.97	0.93	0.96	1.00	0.96	0.99

Table S4 Statistics of pesticides extracted from AgroSAR sets

Sets	Properties	5% Quantile	95% Quantile	Median	Mean	SD[a]
Pesticide	MW	228.3	553.3	354.8	370.1	108.2
	LogP	1.2	7.2	4.1	4.2	1.8
	HBA	1	7	3	3.3	2
	HBD	0	2	0	0.5	0.8
	ROT	2	11	6	6.1	3.1
	arR	0	3	2	1.8	1
Herbicides	MW	225.2	430.7	320.8	324.3	67.7
	LogP	0.8	5.4	3.4	3.3	1.4
	HBA	1	7	3	3.4	1.8
	HBD	0	2	1	0.7	0.7
	ROT	2	10	5	5.6	2.4
	arR	0	3	1	1.4	0.8
Insecticides	MW	245.3	608.6	384	403.5	123.4
	LogP	1.4	7.7	4.7	4.6	1.9
	HBA	1	7	3	3.1	2.2
	HBD	0	2	0	0.6	0.8
	ROT	2	11	6	6.4	2.8
	arR	0	3	2	1.8	1
Fungicides	MW	216.3	480.8	336.3	343.6	84.7
	LogP	1.2	6.4	3.8	3.8	1.6
	HBA	1	7	3	3.4	1.7
	HBD	0	2	0	0.5	0.7
	ROT	2	12	5	5.8	3.3
	arR	0	3	2	1.8	1

[a] SD standard deviation

Table S5 Percentages of marketed pesticides used to develop QEPest-SFs passing rule-based filters

Class	Number of pesticides	Lipinski	Tice [1]	Hao [2]
Herbicides	585	97.61 %	64.44 %	-
Insecticides	495	88.46 %	65.79 %	-
Fungicides	278	92.75 %	-	-
Pesticides	1319	93.71 %	-	77.35 %

accurate ClogP values were computed for 78.7% of the pesticides

Table S6 QEPest scores assign to AgroSAR sets at cumulative frequency (CF) values of 0.25, 0.5, 0.75

Scoring function	CF 0.25	CF 0.5	CF 0.75
QEH	0.859	0.72	0.595
QEI	0.746	0.574	0.404
QEF	0.733	0.601	0.39
QEP_{max}	0.835	0.696	0.515
QEP_{avg}	0.734	0.600	0.419

Table S7 Area under the receiver operating curve (AUC)[3] and standard deviation (SD) values achieved by the QEPest scoring functions

Scoring function	QEH	QEI	QEF	QEP_{max}	QEP_{avg}
AUC (±SD)	0.721 (±0.007)	0.668 (±0.003)	0.677 (±0.003)	0.643 (±0.002)	0.651 (±0.002)

Table S8 Optimum cutoffs for QEPest-SFs as suggested by Matthews correlation coefficients and sensitivity-specificity *versus* cutoff (Se-Sp Cutoff) plots

Metric	QEH	QEI	QEF	QEP_{max}	QEP_{avg}
Matthews correlation coefficients	0.608	0.784	0.607	0.702	0.639
Se-Sp Cutoff	0.650	0.500	0.510	0.630	0.530

Table S9 Standardization of pesticides before molecular properties generation;

Number	Standardization step
1	Remove fragment
2	Strip salts
3	Remove water
4	Transform nitro
5	Transform nitroso
6	Transform Azide
7	Transform Isocyanate
8	Transform Aromatic N-Oxide
9	Transform Tertiary N-Oxide
10	Transform Phosphoric
11	Transform Phosphonium Ylide
12	Transform Sulfoxide
13	Transform Sulfon
14	Disconnect Metal Atoms
15	Neutralize
16	Clean Isotones
17	Tautomerize

ChemAxon's Standardizer module - JChem 6.0.0, 2013, ChemAxon, http://www.chemaxon.com

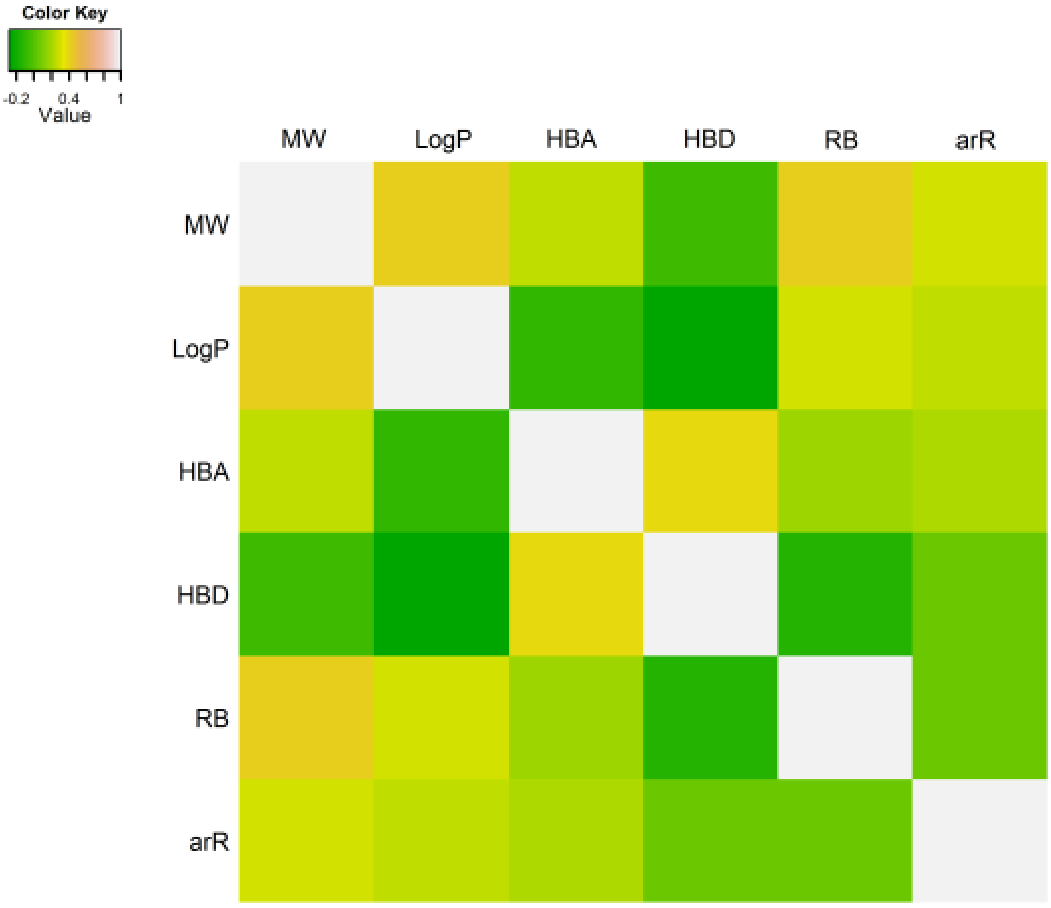

Figure S1 Kendall *tau* [4] values describing the pair wise correlation between MW, LogP, HBA, HBD, RB and arR, in the marketed pesticide set (see paper)

a)

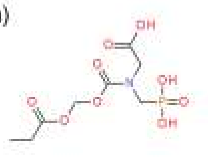

10 (QEH=0.116)

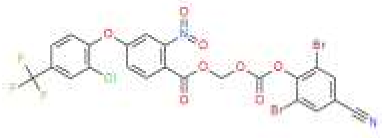

11 (QEH=0.105)

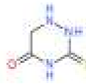

12 (QEH=0.102)

b)

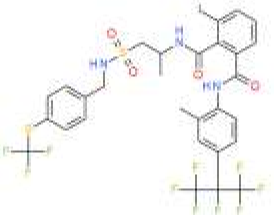

13 (QEI=0.056)

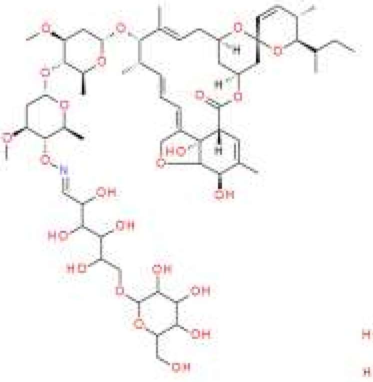

14 (QEI=0.045)

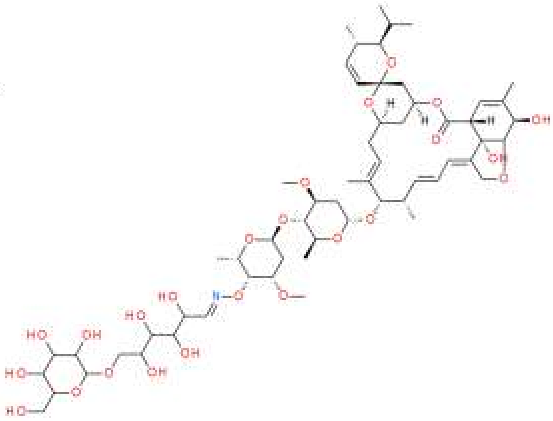

15 (QEI=0.044)

c)

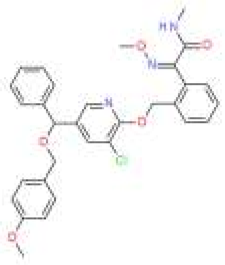

16 (QEF=0.057)

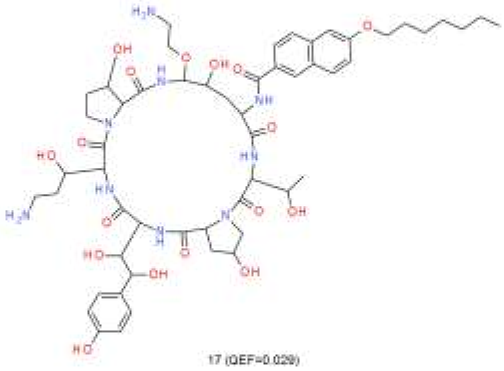

17 (QEF=0.029)

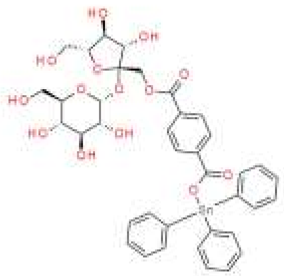

18 (QEF=0.026)

Figure S2 Three of the lowest scored herbicides (a), insecticides (b) and fungicides (c) from AgroSAR dataset (ignoring zero scored representatives)

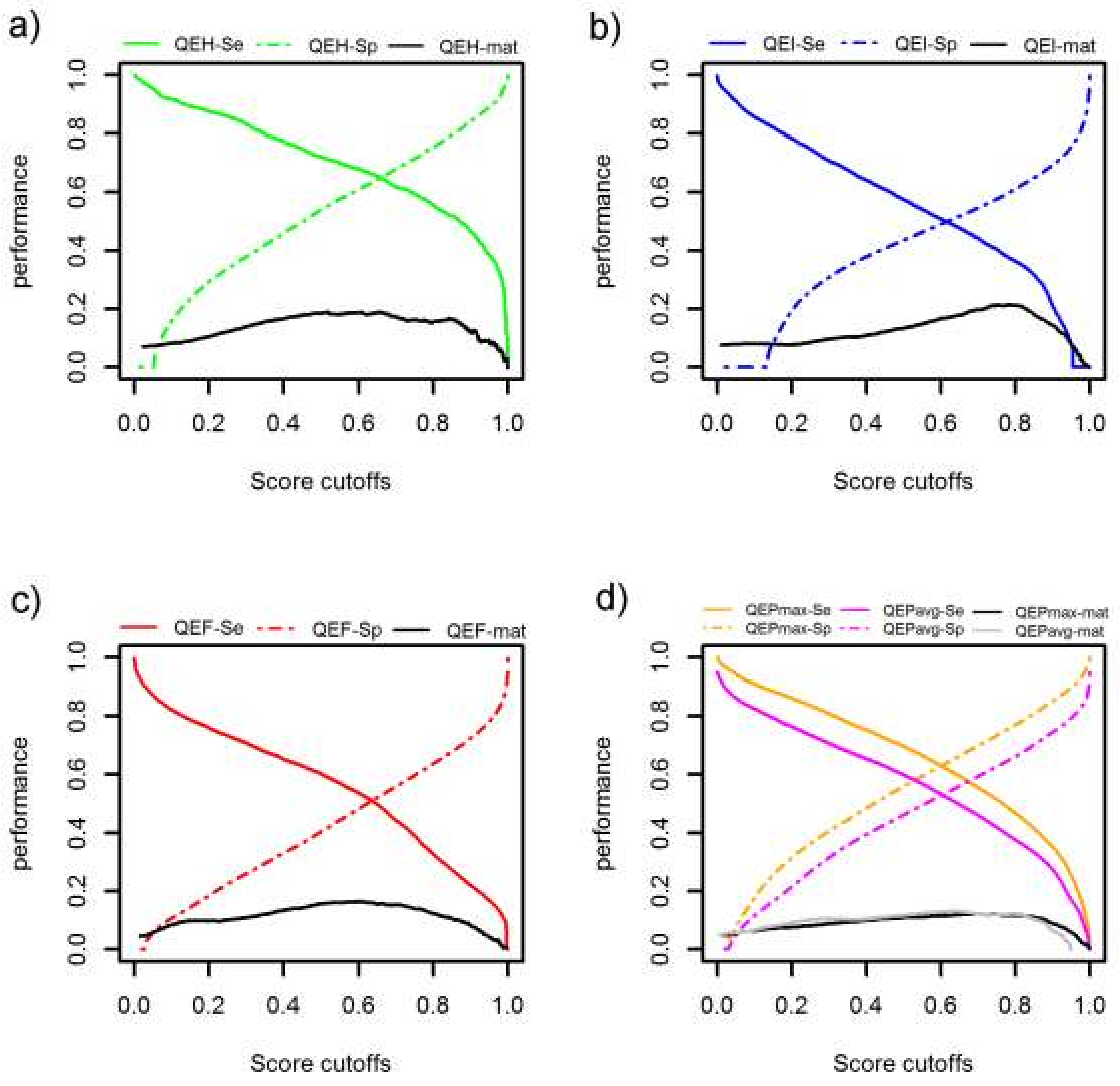

Figure S3 Sensitivity(Se)-specificity(Sp) and Matthews correlation coefficient *versus* score cutoffs plots of AgroSAR (a) herbicides, (b) insecticides, (c) fungicides, (d) pesticides

References

1. Tice CM (2001) Selecting the right compounds for screening: does Lipinski's Rule of 5 for pharmaceuticals apply to agrochemicals? Pest Manag Sci 57: 3–16.

2. Hao G, Dong Q, Yang G (2011) A Comparative Study on the Constitutive Properties of Marketed Pesticides. Mol Inform 30: 614–622.

3. Hanley A, Mcneil J (1982) The meaning and use of the area under a Receiver Characteristic (ROC) curve. Radiology 143: 29–36.

4. Kendall MG (1976) Rank Correlation Methods. 4th Ed. Griffin, London.
