# Supplementary figures and images for: Quantitative estimation of pesticide-likeness for agrochemical discovery
Source: J Cheminform. 2014 Sep 12;6:42. doi: 10.1186/s13321-014-0042-6 (PMC4173135; doi:10.1186/s13321-014-0042-6)

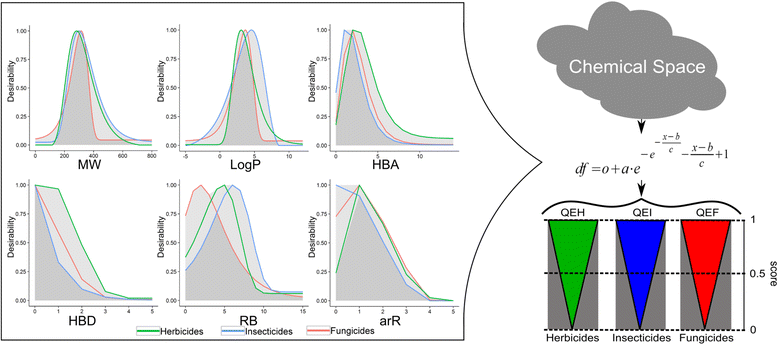

Supplement: Supplementary file 3 — Authors’ original file for figure 1 [file 13321_2014_42_MOESM3_ESM.gif]

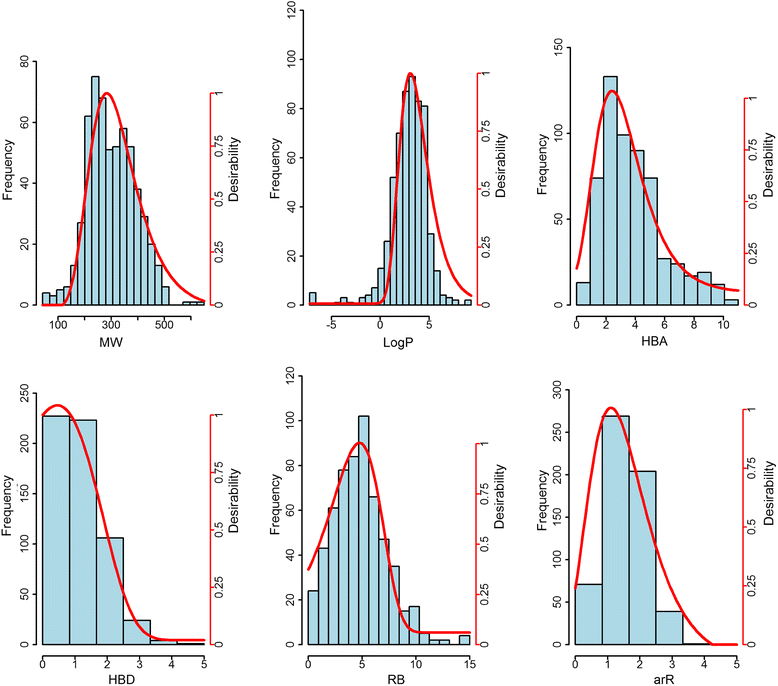

Supplement: Supplementary file 4 — Authors’ original file for figure 2 [file 13321_2014_42_MOESM4_ESM.gif]

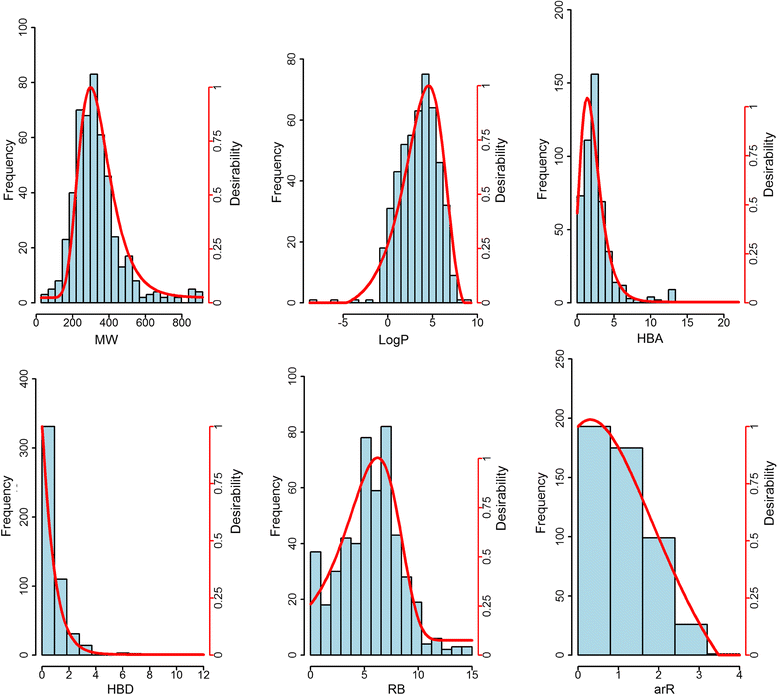

Supplement: Supplementary file 5 — Authors’ original file for figure 3 [file 13321_2014_42_MOESM5_ESM.gif]

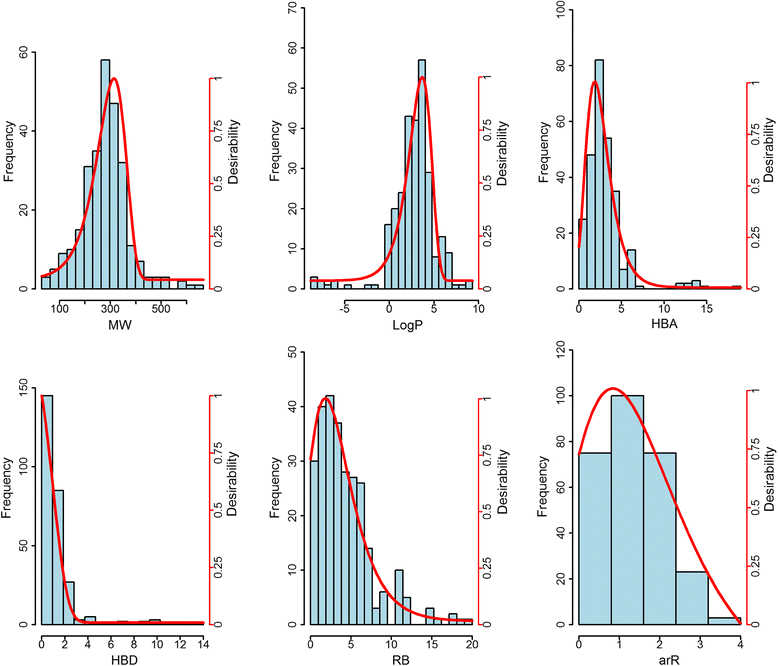

Supplement: Supplementary file 6 — Authors’ original file for figure 4 [file 13321_2014_42_MOESM6_ESM.gif]

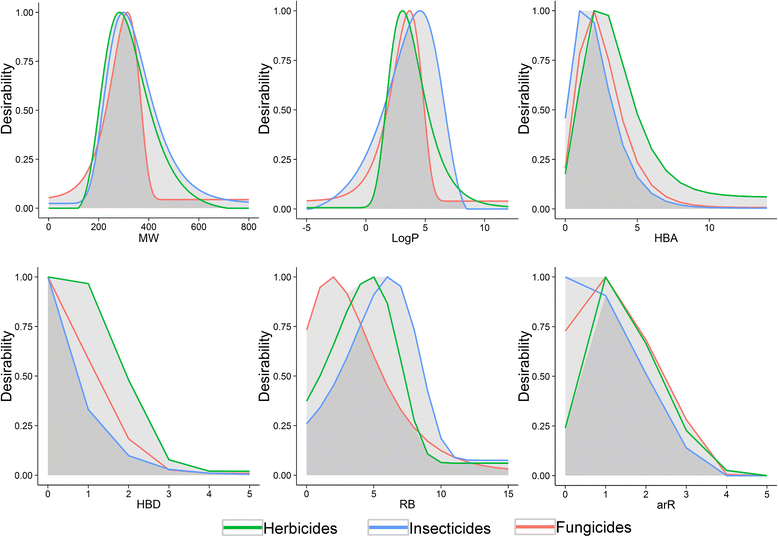

Supplement: Supplementary file 7 — Authors’ original file for figure 5 [file 13321_2014_42_MOESM7_ESM.gif]

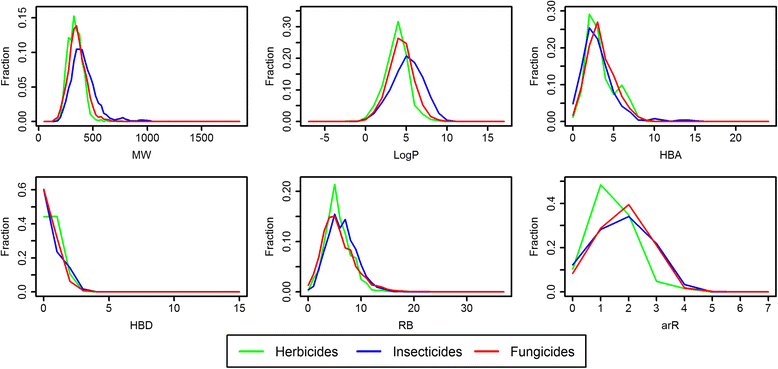

Supplement: Supplementary file 8 — Authors’ original file for figure 6 [file 13321_2014_42_MOESM8_ESM.gif]

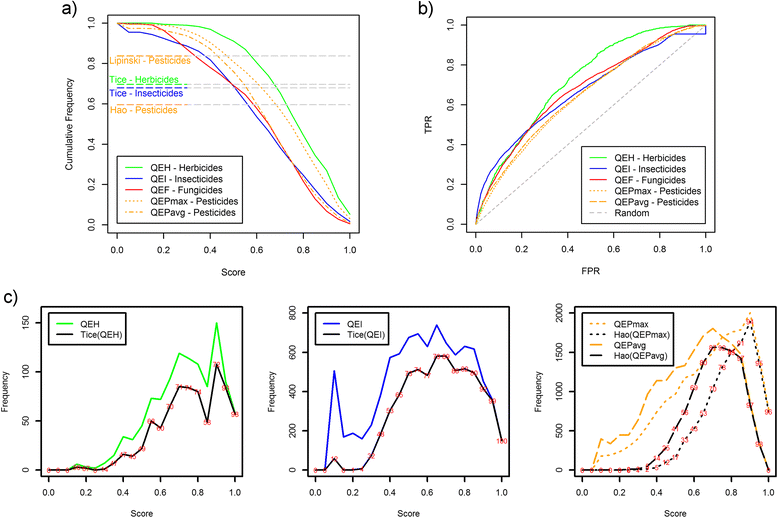

Supplement: Supplementary file 9 — Authors’ original file for figure 7 [file 13321_2014_42_MOESM9_ESM.gif]

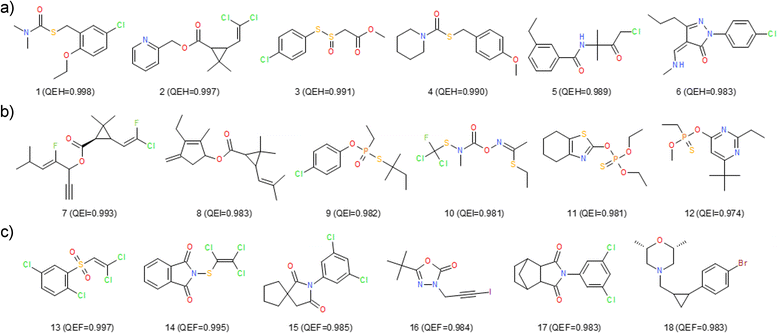

Supplement: Supplementary file 10 — Authors’ original file for figure 8 [file 13321_2014_42_MOESM10_ESM.gif]
